# Inducible nitric oxide synthase deficiency promotes murine-β-coronavirus induced demyelination

**DOI:** 10.1186/s12985-023-02006-1

**Published:** 2023-03-25

**Authors:** Mithila Kamble, Fareeha Saadi, Saurav Kumar, Bhaskar Saha, Jayasri Das Sarma

**Affiliations:** 1 Department of Biological Sciences, Indian Institute of Science Education and Research Kolkata, Mohanpur, 741246 India; 2 National Centre for Cell Science, Ganeshkhind, Pune, India; 3 Department of Ophthalmology, University of Pennsylvania Scheie Eye Institute, Philadelphia, PA 19104 USA

**Keywords:** NOS2, Mouse Hepatitis Virus (MHV), RSA59, Neuroinflammation, Demyelination, Phagocytic MG/Mφ, CD206, TREM2, Arg1

## Abstract

**Background:**

Multiple sclerosis (MS) is characterized by neuroinflammation and demyelination orchestrated by activated neuroglial cells, CNS infiltrating leukocytes, and their reciprocal interactions through inflammatory signals. An inflammatory stimulus triggers inducible nitric oxide synthase (NOS2), a pro-inflammatory marker of microglia/macrophages (MG/Mφ) to catalyze sustained nitric oxide production. NOS2 during neuroinflammation, has been associated with MS disease pathology; however, studies dissecting its role in demyelination are limited. We studied the role of NOS2 in a recombinant β-coronavirus-MHV-RSA59 induced neuroinflammation, an experimental animal model mimicking the pathological hallmarks of MS: neuroinflammatory demyelination and axonal degeneration.

**Objective:**

Understanding the role of NOS2 in murine-β-coronavirus-MHV-RSA59 demyelination.

**Methods:**

Brain and spinal cords from mock and RSA59 infected 4–5-week-old MHV-free C57BL/6 mice (WT) and NOS2-/- mice were harvested at different disease phases post infection (p.i.) (day 5/6-acute, day 9/10-acute-adaptive and day 30-chronic phase) and compared for pathological outcomes.

**Results:**

NOS2 was upregulated at the acute phase of RSA59-induced disease in WT mice and its deficiency resulted in severe disease and reduced survival at the acute-adaptive transition phase. Low survival in NOS2-/- mice was attributed to (i) high neuroinflammation resulting from increased accumulation of macrophages and neutrophils and (ii) Iba1 + phagocytic MG/Mφ mediated-early demyelination as observed at this phase. The phagocytic phenotype of CNS MG/Mφ was confirmed by significantly higher mRNA transcripts of phagocyte markers-CD206, TREM2, and Arg1 and double immunolabelling of Iba1 with MBP and PLP. Further, NOS2 deficiency led to exacerbated demyelination at the chronic phase as well.

**Conclusion:**

Taken together the results imply that the immune system failed to control the disease progression in the absence of NOS2. Thus, our observations highlight a protective role of NOS2 in murine-β-coronavirus induced demyelination.

**Supplementary Information:**

The online version contains supplementary material available at 10.1186/s12985-023-02006-1.

## Background

Multiple sclerosis (MS) is the most widely studied neuroinflammatory and demyelinating disorder of the CNS and is common among young adults. It is characterized by the destruction of myelin and myelin-producing oligodendrocytes. The disease etiology is associated with autoimmunity, several genetic and environmental factors, and pathogens such as viruses. A recent groundbreaking study by Bjornevik et al. [1] suggested Epstein Barr virus (EBV) as one of the leading causes of MS confirming the involvement of virus etiology. Neuroinflammation in MS is mediated by the production of several inflammatory mediators, including cytokines, chemokines, and reactive oxygen and nitrogen species by activated glial cells, such as microglia and astrocytes, and CNS infiltrating myeloid and lymphoid cells [2, 3]. In response to an inflammatory stimulus, inducible nitric oxide synthase (iNOS or NOS2) catabolizes L-arginine to generate nitric oxide (NO) that has been reported to significantly contribute to the oligodendrocyte degeneration, demyelination, and neuronal damage in MS [4].

During inflammation, NOS2 is induced in all leukocytes, including macrophages, dendritic cells, NK cells, primary tumor cells, and to a certain extent, activated T cells [5]. NOS2 expression by microglia and macrophages is a prominent pro-inflammatory marker of neuroinflammation and is implicated in MS pathophysiology [4].

MS patients revealed NOS2 expression in the chronic active plaques in diverse cell types, including microglia/macrophages (MG/Mφ), ependymal cells, inflammatory cells, and to a certain extent astrocytes [6]. Studies using experimental animal models of MS have shown mixed results. Several experiments report improved disease symptoms upon inhibition of NOS2 or NOS2 deficiency [7–12], while in others, it rendered the system vulnerable to pathogen attack or exacerbated the disease [13–16]. Though studies implicating NOS2 in demyelination are limited, most indicate no effect or a detrimental effect of NOS2.

In this study, we investigated the effect of NOS2 on the pathological outcome of neuroinflammation and neuroinflammatory demyelination induced by RSA59, a spike gene recombinant strain of murine β-coronavirus (MHV-A59), in an effort to better understand the NOS2-mediated pro-inflammatory immune response [17]. RSA59 infection induces a biphasic disease in mice characterized by acute phase (day 5/6 post-infection [p.i.]) hepatitis, meningitis, encephalitis, and successive chronic phase (day 15–30 p.i.) demyelination and concomitant axonal loss [17–22]. We previously showed an interplay between MG/Mφ and CD4 + T cells via CD40 receptor (CD40) and CD40 ligand (CD40L) interaction in the RSA59 induced model. The absence of either CD4 + T cells or CD40L impaired viral clearance from the CNS and aggravated the severity of demyelination in the chronic phase [23, 24]. Confirming evidence of the protective role of CD4 + T cells was further illustrated in a parallel study performed in Ifit2-/- mice. RSA59 infected Ifit2-/- mice presented with dampened activation of MG/Mφ resulting in low recruitment of CD4 + T cells in the CNS, which led to severe clinical disease and high mortality in the mice [25]. Having studied the adaptive arm and its crosstalk with the innate arm during the RSA59-induced immune response, we next examined the role of innate immune MG/Mφ to investigate the NOS2-mediated proinflammatory immune response. We have compared 4–5-week-old male C57BL/6 mice with NOS2-/- mice for pathological outcomes on RSA59 infection.

In the current study, we showed that NOS2 expression was upregulated at the acute phase of RSA59 infection and its absence led to more severe clinical disease score as compared to WT mice. Most strikingly NOS2-/- mice exhibited a drop in survival at the acute-adaptive transition phase (day 9/10 p.i.) associated with significant increase in the infiltration of macrophages and neutrophils in the brain. Resultantly, the transcript levels of TNFα, IFNγ and Ifit2 were higher in the NOS2-/- brains. However, the virus was successfully cleared from the CNS in both NOS2-/- and wildtype mice. In addition, demyelination pathology was observed as early as day 9/10 p.i. in infected NOS2-/- mice as compared to day 30 p.i. in the WT infected mice. The demyelinating plaques showed accumulation of amoeboid phagocytic MG/Mφ which was further confirmed by significant upregulation of phagocytic markers- CD206, TREM2 and Arginase1 in the CNS. Confocal imaging of spinal cords at day 9/10 p.i. showed engulfment of myelin basic protein (MBP) and proteolipid protein (PLP) by Iba1 + MG/Mφ. At the chronic phase as well NOS2-/- mice showed exacerbated demyelination. The results together indicated a protective role of NOS2 against murine β-coronavirus-induced demyelination.

## Materials and methods

### Virus, inoculation of mice, and experimental design

Wild-type C57BL/6 mice (Jackson Laboratory, B6.129P2-tm1Lau/J) were obtained from Jackson Laboratory and NOS2-/- mice (Jackson Laboratory, B6.129P2-tm1Lau/J, Stock No. 002609) [26] were obtained from the Animal Facility at the National Centre of Cell Science Pune, India. NOS2-/- mice did not show any inherent pathological differences to the WT mice (Additional file 1: Fig S4 and S5). Five-week-old male MHV-free WT and NOS2-/- mice were infected with RSA59, an isogenic recombinant strain of the demyelinating mouse hepatitis virus; MHV-A59, described previously [27]. The mice were inoculated intracranially with RSA59 at 20,000 (50% of 50% lethal dose, LD50) and 10,000 PFUs. The requirement of 10,000 PFUs dose was due to low survival of the NOS2-/- mice infected with 20,000 PFUs of RSA59. Mock infected controls were inoculated with phosphate-buffered saline (PBS) plus 0.75% bovine serum albumin (BSA).

Mice were monitored daily post-infection for disease signs and symptoms, and changes in their weight were noted. Clinical disease severity was graded using the following scale: 0: no disease symptoms; 0.5: ruffled fur; 1: hunched back with mild ataxia; 1.5: hunched back with mild ataxia and hind limb weakness; 2: ataxia, balance problems and or partial paralysis; 2.5: one leg completely paralyzed, motility issues but still able to move around with difficulty; 3: severe hunching/wasting/paralysis of both hind limbs and severely compromised mobility; 3.5: severe distress, complete paralysis, and moribund; 4: dead. For RNA studies, mice were sacrificed on day 6 (acute phase), day 10 (acute-adaptive transition phase), day 15 (chronic adaptive phase), and day 30 p.i. (chronic phase). For histopathological, immunohistochemical analyses, viral titer estimation, and flow cytometry experiments, mice were sacrificed at day 9/10 p.i. (acute-adaptive chronic phase) and day 30 p.i. (chronic phase).

### Estimation of viral replication

WT and NOS2-/- mice infected with RSA59 at 10,000 PFUs and 20,000 PFUs were sacrificed at day 9/10 p.i. Brains were harvested and placed into 1 ml of isotonic saline containing 0.167% gelatin (gel saline). Brain tissues were weighed and kept frozen at − 80 °C until titered. Tissues were subsequently homogenized and using the supernatant, viral titers were quantified by standard plaque assay protocol on tight monolayers of L2 cells as described previously [27] using the formula: plaque-forming units (PFUs) = (no. of plaques × dilution factor/ml/gram of tissue) and expressed as log10 PFUs/gram of tissue.

### Flow cytometry analysis

Mice were transcardially perfused with 20 ml PBS, and half brains were homogenized in 2 ml of RPMI containing 25 mM HEPES (pH 7.2), using Tenbroeck tissue homogenizers. Tissue homogenate was then centrifuged at 450 g for 10 min. Obtained cell pellets were resuspended in RPMI containing 25 mM HEPES, adjusted to 30% Percoll (Sigma), and underlaid with 1 ml of 70% Percoll. A second centrifugation cycle was applied at 800 g for 30 min at 4 °C, following which the cells were recovered from the 30–70% interface, washed with RPMI, and suspended in FACS buffer (0.5% bovine serum albumin in Dulbecco’s PBS). Specific cell types were identified by staining with fluorochromes like fluorescein isothiocyanate (FITC), phycoerythrin [(PE), (PECy7)], peridinin chlorophyll protein [(PerCP), (PerCpCy5.5)], allophycocyanin [(APC), (APCCy7)] and violet excitable dyes [(V450), (V500)] conjugated MAb for 45 min on ice in FACS buffer. Expression of surface markers was characterized with MAb (all from BD Biosciences except where otherwise indicated) specific for CD45 (clone Ly-5), CD11b (clone M1/70), CD11c (clone HL3), CD3 (clone 145-2C11), CD4 (clone GK1.5), CD8 (clone 53–6.7), NK1.1 (clone PK136), CD25 (clone PC61), and Foxp3 (clone G155-178). Intra-nuclear staining was performed for Foxp3 after fixation and permeabilization (BD 562574) per the manufacturer’s guidelines*.*

To detect the expression of NOS2 in the immune cell subsets, intracellular staining for NOS2 was performed following the staining of surface markers as mentioned above. The cells were fixed and permeabilized (BD 554714) as per the manufacturer’s guidelines and stained with anti-NOS2 antibody (Invitrogen PA3-030A) which was followed by washing and subsequent staining with secondary antibody (GαR APC).

Samples were acquired on a BD FACSVerse flow cytometer (BD Biosciences) and analyzed on FlowJo 10 software (Treestar, Inc., Ashland, OR) [25]. First, doublet exclusion using FSC‐A and FSC‐W was performed, and then cells were gated based on forward scatter (FSC), and side scatter (SSC) to focus on live cells. Further, the cells were analyzed to differentiate myeloid and lymphoid populations. Myeloid cells were gated from a primary gating on CD45, and an independent CD3 gating or CD3 gating in addition to CD45 was applied for the lymphoid populations. Single colors and FMOs were used in all the experiments. Beads were gated based on their FSC/SSC pattern.

### Histopathology and immunohistochemical analysis

Mice were sacrificed at day 9/10 and day 30 p.i. Following transcardial perfusion with PBS and post-fixation with 4% paraformaldehyde for 26–48 h, spinal cord tissues were harvested and embedded in paraffin. For this, sections from cervical, lumbar, and thoracic regions of spinal cord from one biological sample were embedded in a single mould and sectioned into 5 μm thick transverse sections using Thermo Scientific HM 355 S sectioning system. The sections were then prepared and stained with hematoxylin and eosin for histopathologic analysis. Luxol fast blue (LFB) staining was performed to evaluate demyelination in the spinal cord tissues, as described previously with minor modifications [23]. Immunohistochemistry was performed in spinal cord section using antibody directed against MG/Mφ-specific calcium-binding protein; Ionized calcium-binding adaptor molecule 1 (Iba1) (Wako; Catalog No. 019–19,741) in 1:600 dilution. Bound primary antibodies were detected by an avidin–biotin immunoperoxidase technique (Vector Laboratories) using 3,3-diaminobenzidine (DAB) as the substrate [28]. Control slides from mock infected mice were stained in parallel. All the slides were coded and read in a blinded manner.

### Quantification of histopathological sections

Image analysis was performed using Fiji’s basic densitometric thresholding application (Image J, NIH Image, and Scion Image) as described previously [29]. Briefly, image analysis for Iba1 stained sections was performed by capturing the images at the × 4 for brain and × 10 for the spinal cord so that the entire section (i.e., the scan area) could be visualized within a single frame. The RGB image was deconvoluted into three different colors to separate and subtract the DAB-specific staining from the background hematoxylin staining. The perimeter of each spinal cord tissue was digitally outlined, and the area was calculated in square micrometers. A threshold value was fixed for each image to ensure that all antibody-marked cells were taken into consideration. The amounts of Iba1 staining were termed the percent area of staining which was calculated for every single section available per spinal cord and plotted.

To determine the area of demyelination, LFB stained spinal cord transverse-sections from each mouse were chosen and analyzed using Fiji (Image J, NIH Image, and Scion Image) [29, 30]. The total perimeter of the white matter regions in each transverse-section was marked, and the area was calculated by adding together the dorsal, ventral, and anterior white matter areas in each section. Also, the total area of the demyelinated regions was outlined and collated for each section separately. The percentage of spinal cord demyelination per section per mouse was calculated for all available sections.

### RNA isolation, reverse transcription, and quantitative PCR

RNA was extracted from brain tissues of RSA59 infected WT and NOS2-/- mice and mock infected mice using the TRIzol isolation protocol following transcardial perfusion with diethylpyrocarbonate (DEPC)-treated PBS. The total RNA concentration was measured using a NanoDrop ND-2000 spectrophotometer. One microgram of RNA was used to prepare cDNA using a high-capacity cDNA reverse transcription kit (Applied Biosystems). Quantitative real-time PCR analysis was performed using a DyNAmo ColorFlash SYBR Green qPCR kit (Thermo Scientific) in a Step One Plus real-time PCR system (Thermo Fisher Scientific) under the following conditions: initial denaturation at 95 °C for 7 min, 40 cycles of 95 °C for 10 s and 60 °C for 30 s and melting curve analysis at 60 °C for 30 s. Reactions were performed in duplicates or triplicates. Sequences for the primers used are given in Table 1. Absolute quantitation was achieved using the threshold (ΔCT) method. mRNA expression levels of target genes in mock and RSA59 infected WT and NOS2-/- mice were normalised with GAPDH. The average of the replicates for each mouse biological sample were expressed as mRNA expression to depict the changes between the infected WT and NOS2-/- groups as well as between the mock infected WT and NOS2-/- groups, if any.

**Table 1:** List of primers used for quantitative real time PCR

	Primer sequence (5′- 3′)	
	Forward primer	Reverse primer
GAPDH	GCCCCTTCTGCCGATGC	CTTTCCAGAGGGGCCATCC
NOS2	TGGCCACCTTGTTCAGCTACGC	GGTGCCAAGGCCAAACACAGCATA
N gene	GTTGCAAACAGCCAAGCG	GGGCGCAAACCTAGT
TNFα	CTGTAGCCCACGTCGTAGC	TTGAGATCCATGCCGTTG
IFNγ	GTCTCTTCTTGGATATCTGGAGGAACT	GTAGTAATCAGGTGTGATTCAATGACGC
Ifit2	GGGAAAGCAGAGGAAATCAA	TGAAAGTTGCCATACAGAAG
CD206	TGGAGGCTGATTACGAGCAGT	TTGGTTCACCGTAAGCCCAAT
P2Y6	TGCCAATCTACATGGCAGCA	CACGACTCCACACACTACCC
TREM2	CAGTGTCAGAGTCTCCGAGG	CACAGGATGAAACCTGCCTGG
Arg1	CATGGAAGTGAACCCAACTC	CTGTCTGCTTTGCTGTGATG

### Immunofluorescence microscopy

Infected WT and NOS2-/- mice were sacrificed at day 9/10 p.i. and day 30 p.i. and spinal cord tissue sections were harvested, processed, and embedded in paraffin. 5 μm thick transverse sections of spinal cord were used to perform immunofluorescence imaging. The tissue sections parallel to those used for corresponding histopathology were stained. Briefly, the slides were deparaffinized followed by rehydration and antigen unmasking. The slides were then permeabilized with 0.2% Triton X-100 in phosphate buffered saline (PBS) by shaking incubation at RT for 15 min and blocked using 1% bovine serum albumin (BSA) prepared in 0.2% Triton X-100-1X PBS solution at 37 °C for 1 h. This was followed by shaking incubation at 4 °C for 16 h with primary antibodies prepared in blocking solution. Each section on each sample slide was stained for the following combinations separately: i) Anti-Iba1 (1: 500, Wako; Catalog No. 019–19,741) and anti-MBP home-made serum antibody (1:1); ii) Anti-Iba1 (1:500) and home-made serum antibody against PLP (1:1). Slides were then washed and incubated for 1 h at 37 °C with combination of secondary antibodies AlexaFluor568 and AlexaFluor488 (1:750 each) prepared in blocking solution. Finally, the slides were washed in PBS and mounted using Vectashield with DAPI.

Epifluorescence images were acquired using Olympus virtual slide scanner (VS200) and processed using OlyVIA software. Confocal imaging was performed using Zeiss Confocal Microscope (LSM710) and the images were processed with Zen 2010 software.

### Statistical analyses

Values were represented as mean values ± standard errors of the mean (SEM). Values were subjected to unpaired student’s t-tests with Welch’s correction or Kruskal- Wallis One- Way ANOVA or Ordinary One- Way ANOVA or Two- Way ANOVA with multiple comparison tests (Tukey’s test and the Holm-Sidak test) for calculating the significance of differences between the means. Log-rank (Mantel-Cox) test was used for calculating the statistical significance in mortality between groups. All statistical analyses were done using GraphPad Prism 8 (La Jolla, CA). A *P*-value of < 0.05 was considered statistically significant.

## Results

### NOS2 is induced in C57BL/6 WT mice during the acute phase (day 5/6 p.i.) of the RSA59 infection and its deficiency results in increased mortality in the infected mice

**Fig. 1:**
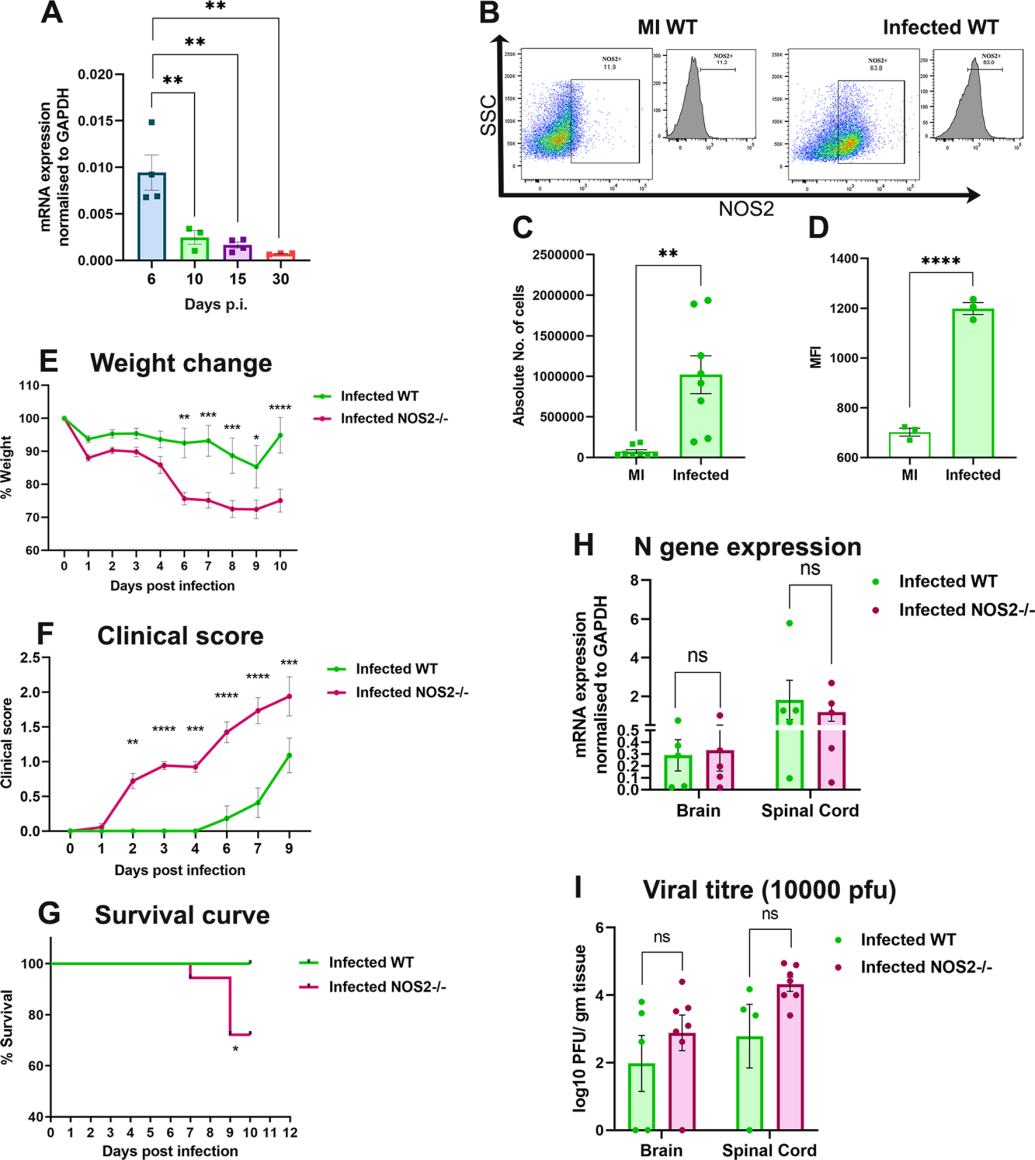
RSA59 infection upregulates NOS2 expression in WT brains at the acute phase (day 5/6 p.i.) and its deficiency results in drop in survival at the acute-adaptive transition phase (day 9/10 p.i.) NOS2 transcripts were determined in the brain of WT mice (n = 3 to 4 per group per time point) infected at 20,000 PFUs at days 6, 10, 15 and 30 p.i. by qRT-PCR. Results were normalised to GAPDH, and values calculated using the ΔCT method were compared and expressed as Mean ± SEM (**A**). NOS2 expression was determined by flow cytometry in the brain harvested from WT mice infected at 10,000 PFUs at day 5/6 p.i. (**B**) shows representative flow cytometry dot plots and histograms showing percentages of NOS2 + cells, gated from live cell populations that were gated from singlets, and their absolute numbers and NOS2 MFI are graphically represented in (**C**) and (**D**), respectively. Wildtype (WT) (n = 11 to 14) and NOS2-/- (n = 17 to 18) mice were infected with 20,000 PFUs of RSA59 and monitored daily for weight change (**E**), development of clinical disease (**F**) and survival (**G**). Clinical scores were assigned by an arbitrary scale of 0–4 as described in Materials and Methods. N gene (viral nucleocapsid gene) transcripts levels in the CNS at day 9/10 p.i. (**H**) were determined by qRT-PCR and values calculated using the ΔCT method were represented graphically as mRNA expression normalised to GAPDH. (n = 3 to 5). Plaque assay was performed to determine viral titers CNS of mice (n = 4 to 7) infected at 10,000 PFUs day 9/10 p.i. (**I**). Statistical analysis was performed using Ordinary One- way ANOVA test for (**A**); unpaired student’s t test with Welch’s correction for (**C**) and (**D**); Two- Way ANOVA with Tukey’s multiple comparison test for (**E**), (**F**), (**H**), and (**I**); and Log-rank (Mantel-Cox) test for survival proportions (**G**). Results were expressed as Mean ± SEM. *Asterisk represents statistical significance between infected WT and infected NOS2-/- groups. *p* < 0.05 was considered as significant. *p < 0.05, **p < 0.01, ***p < 0.001 and ****p < 0.0001. Each dot in the dot plots represents one biological sample

Inflammatory stimuli are major factors that induce NOS2 expression, which is otherwise strictly regulated within the cells [31]. WT mice infected with 20,000 PFUs (50% LD50) of RSA59 showed significantly high expression of NOS2 transcripts at day 5/6 p.i. which is the peak of acute phase neuroinflammation, after which the levels declined (Fig. 1A). Flow cytometry analysis further confirmed the upregulation in NOS2 protein expression in brains of WT mice infected at 10,000 PFUs on day 5/6 p.i. As compared to the mock-infected controls, both frequency of the NOS2-expressing cells- primarily macrophages, natural killer cell, and natural killer T cell population (Additional file 1: Fig S1)- and the intensity of NOS2 expression (MFI) were significantly higher in the brain lysates of infected WT mice (Fig. 1B, C, and D). The data thus proved an innate immune effector function of NOS2 in RSA59-induced acute neuroinflammation.

Further experiments comparing WT and NOS2-/- mice showed that NOS2 deficiency led to significant weight loss (Fig. 1E), heightened clinical score (Fig. 1F) and high mortality in mice infected with 20,000 pfus of RSA59 (Fig. 1G). Despite this, NOS2 deficiency did not affect acute phase CNS pathology (Additional file 1: Fig S2). However, the survival of NOS2 deficient mice dropped significantly at day 9/10 p.i. i.e., at the acute-adaptive transition phase. To check whether the drop in survival could be attributed to incomplete viral clearance, viral load in the CNS was determined. Transcript levels of N (nucleocapsid) gene (Fig. 1H) and viral titers (Fig. 1I) in infected WT and NOS2-/- mice remained comparable at the acute-adaptive transition phase indicating that the heightened disease severity observed in NOS2-/- mice was due to factors intrinsic to the immune responders.

### NOS2 deficiency results in increased infiltration of macrophages and neutrophils in the brain at the acute-adaptive transition phase

**Fig. 2:**
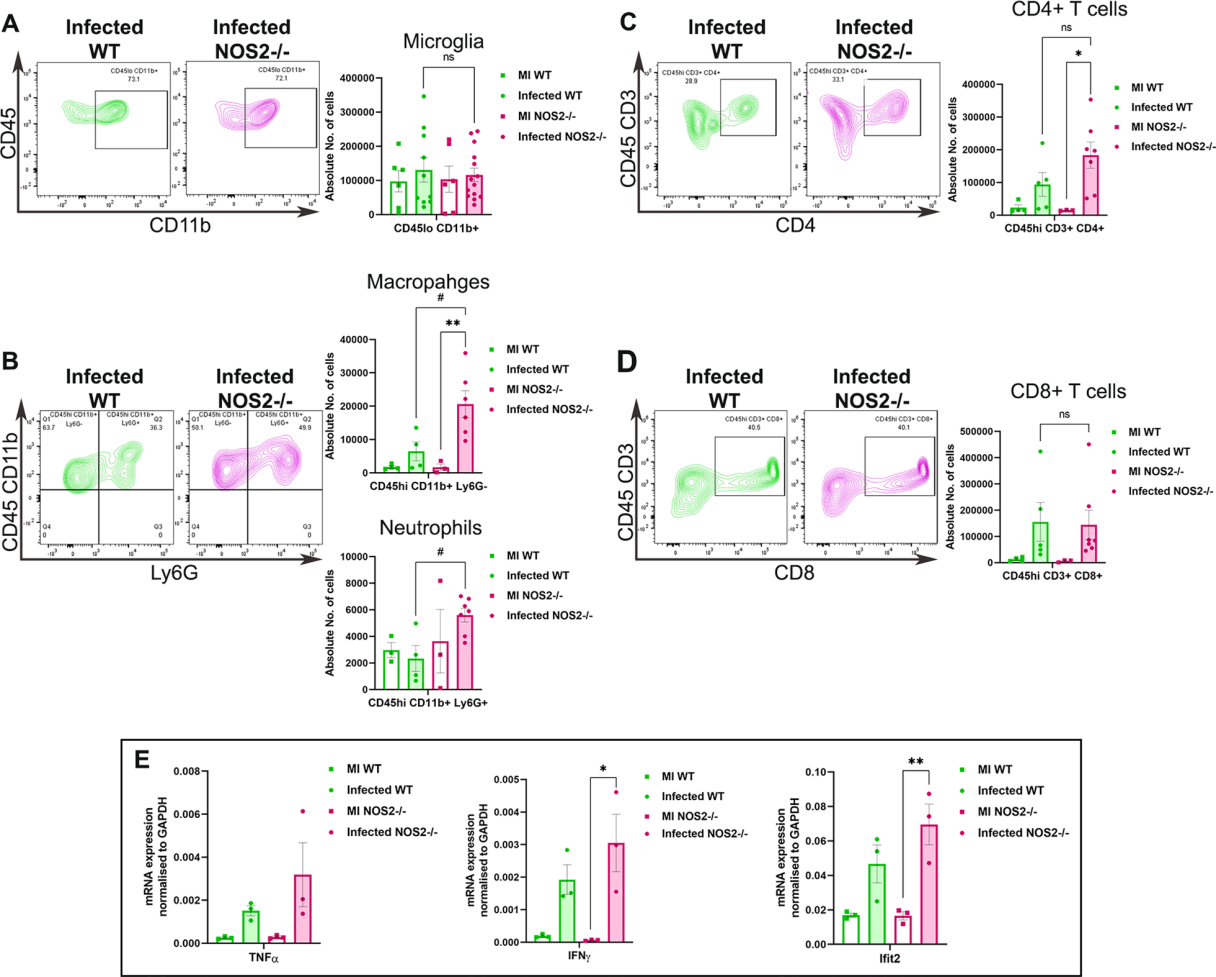
NOS2 deficiency increases the infiltration of macrophages and neutrophils in RSA59 infected mice brains. Flow cytometry analysis was performed on brains from WT and NOS2-/- mock and RSA59 infected (10,000 PFUs) brains at day 9/10 p.i. Infiltrating peripheral cells were distinguished by CD45hi gating from live cell populations gated from singlets. Similarly, CD45lo gate was used to extract brain resident cell populations. Representative flow cytometry contour plots showing percentages of CD45lo CD11b + microglia populations and their graphical representation in mock and RSA59 infected WT and NOS2-/- groups are given in (**A**). Percentages of CD45hi CD11b + Ly6G- macrophages and CD45hi CD11b + Ly6G + neutrophils are presented in representative contour plots and quantification of their numbers in graphical form in (**B**). (**C**) shows the representative contour plots of CD45hi CD3 + CD4 + T cells and its graphical representation of the absolute numbers. Similarly (**D**) shows the representative contour plots of CD45hi CD3 + CD8 + T cells and its graphical representation of the absolute numbers. Transcript levels of TNFα, IFNγ and Ifit2 assessed in WT and NOS2-/- mice brains are shown in (**E**). (Real time quantitative PCR experiment was performed in triplicates and average expression per sample as calculated by ΔCT method was plotted. Each dot corresponds to one biological sample.) Statistical analysis was performed using Two- Way ANOVA with Sidak’s multiple comparison test between mock and infected WT and NOS2-/- groups. Results were expressed as Mean ± SEM. n = 3 to 4 for MI and 5 to 7 for Infected for A, B, C and D. n = 3 for MI and 3 to 4 for Infected for E..*Asterisk represents statistical significance. *p* < 0.05 was considered as significant. *p < 0.05 and**p < 0.01

Previous studies in RSA59 infected mice model have shown that CD4 + T cells and their reciprocal communication with MG/Mφ protected the mice from severe chronic demyelination [23, 24]. NOS2 being a marker of pro-inflammatory MG/Mφ, whether its deficiency affected infiltration of the leukocytes in the CNS at the acute-adaptive transition phase (day 9/10 p.i.) was assessed next. Flow cytometry analysis of WT and NOS2-/- mice infected with 10,000 PFUs showed no differences in the resident CD45loCD11b + microglia (Fig. 2 A). However, there was an increase in the infiltration of the myeloid CD45hiCD11b + Ly6G- macrophages and CD45hiCD11b + Ly6G + neutrophils (Fig. 2 B) in infected NOS2-/- mice brains. The numbers of CD4 + and CD8 + T lymphocytes (Fig. 2 C and D) were comparable in WT and NOS2-/- mice which could explain the adequate viral clearance in NOS2 deficient mice. Whether the inflammatory status of brain was affected due to influx in macrophages and neutrophils was checked next and it was observed that the levels of inflammatory cytokines TNFα, IFNγ and Ifit2 were comparatively higher in infected NOS2-/- mice brains. Thus, NOS2 did not hinder the innate-adaptive crosstalk but there was more influx of macrophages and neutrophils in the brain which influenced the brain inflammatory status.

### NOS2 deficiency results in early severe demyelination and leads to an increased accumulation of phagocytic MG/Mφ in the spinal cords of RSA59 infected mice at the acute-adaptive transition phase

**Fig. 3:**
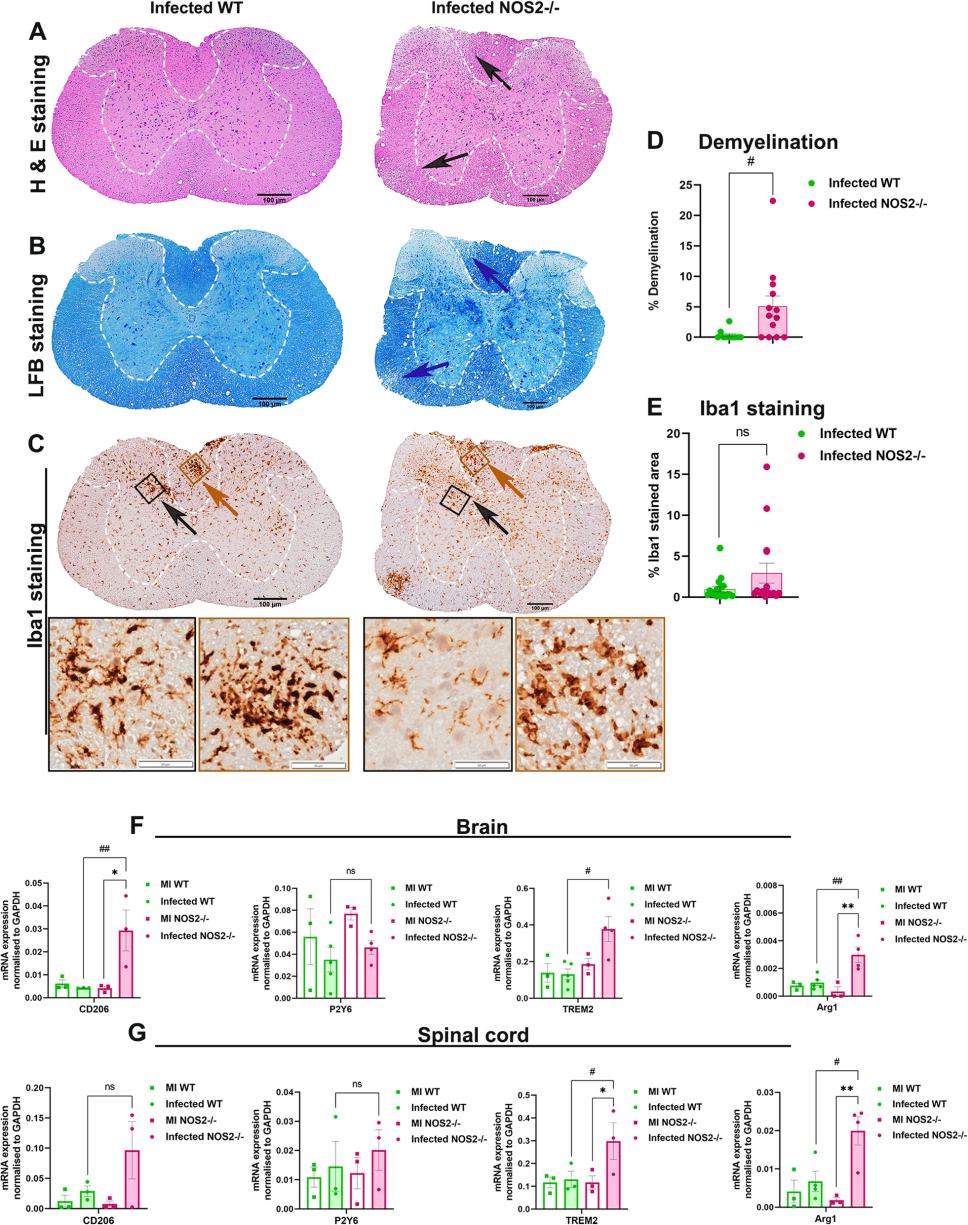
NOS2 deficiency leads to increased demyelination in the spinal cords of RSA59 infected mice at the acute-adaptive transition phase (day 9/10 p.i.) concurrent with increased expression of phagocytic markers. 5 µm thick transverse sections of RSA59 infected (20,000 PFUs) WT and NOS2-/- mice spinal cords were stained for the presence of inflammatory lesions by H & E (**A**), demyelination by LFB (**B**) and activated MG/Mφ by Iba1 (**C**). Black arrows in (**A**) indicate the regions with myelitis. Blue arrows in (**B**) indicate the demyelinating plaques. Black boxed areas highlighted by black arrow represent higher magnification of grey matter area, and brown boxed areas highlighted by brown arrow represent higher magnification of white matter below the corresponding Iba1 stained spinal cord transverse-sections in (**C**). Scale bar 100 µm. (**D**) and (**E**) are the quantification of percent demyelination and percent Iba1 stained area, respectively (n = 4 to 5 mice per group. 1 to 6 sections corresponding to different regions of the spinal cord were analysed and each dot in the graph represent one section from one spinal cord). Results were expressed as Mean ± SEM. *Asterisk represents statistical significance calculated between infected WT and infected NOS2-/- mice using unpaired student’s t test with Welch’s correction. *p* < 0.05 was considered as significant. *p < 0.05. mRNA was isolated from brain and spinal cord tissue of mock and infected (20,000 PFUs) RSA59 WT and NOS2-/- mice. Transcript levels of CD206, P2Y6, TREM2 and Arg1 were analyzed for brain (**F**) and spinal cord (**G**) by qRT- PCR. Results were expressed as Mean ± SEM. (n = 3 to 5. The mRNA expression was calculated by ΔCT method and average expression per biological sample was plotted.)) *Asterisk represents statistical significance using Two- ANOVA with Tukey’s multiple comparisons test. *p* < 0.05 was considered as significant. *p < 0.05 and **p < 0.01

Next, the effect of the increased number of macrophages and neutrophils and thereby the altered inflammatory status of the brain on CNS pathology was checked. Although brain pathology was not affected (Additional file 1: Fig S3), pathology of the spinal cord in WT and NOS2-/- mice showed drastic differences on day 9/10 p.i. (acute-adaptive transition phase). H&E staining of the spinal cord sections showed higher myelitis in infected NOS2-/- mice as compared to WT controls (Fig. 3 A, black arrows). Additionally, Luxol fast blue staining (LFB) revealed increased demyelination in the NOS2-/- mice (Fig. 3 B, dashed lines indicated by blue arrows). The demyelinating plaques showed a distinct accumulation of Iba1 + MG/Mφ. Quantification of the percent demyelination and Iba1 expression in the spinal cords of infected mice revealed significantly high demyelination in the absence of NOS2 (Fig. 3D) but a similar expression of Iba1 by the activated MG/Mφ (Fig. 3E). The distinct amoeboid morphology of the Iba1 + MG/Mφ present in the demyelinating plaques in NOS2-/- mice indicated a phagocytic phenotype.

Therefore, to verify the MG/Mφ phenotype status, the transcript levels of different phagocytic markers were assessed at day 9/10 p.i. in WT and NOS2-/- mice infected with 20,000 PFUs. Results showed the transcript levels of CD206, TREM2 and Arg1 were significantly upregulated in brain, TREM2 and Arg1 were significantly upregulated in the spinal cord. while P2Y6 showed no change in both brains (Fig. 3F) and spinal cords of infected NOS2-/- mice (Fig. 3G).

### Absence of NOS2 leads to demyelination due to active engulfment of MBP and PLP by Iba1 + MG/Mφ as early as day 9/10 p.i.

**Fig. 4:**
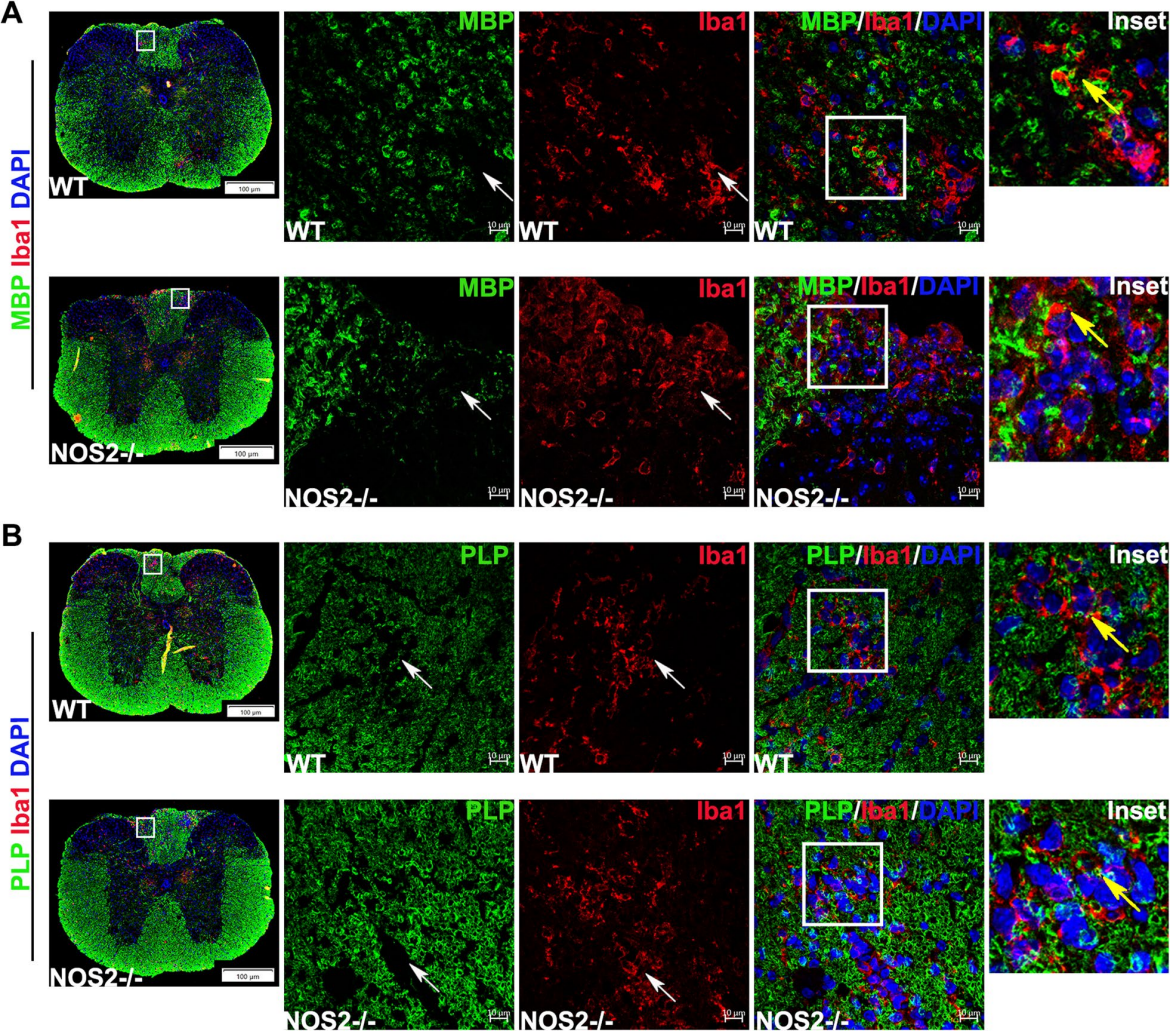
Early demyelination in the absence of NOS2 is an effect of engulfment of MBP and PLP by Iba1 + MG/Mφ. (**A**) to (**B**) are representative epifluorescence scans of spinal cord sections and respective confocal photomicrographs. 5 μm thick transverse sections of paraffin embedded spinal cords from infected WT and NOS2-/- mice at day 9/10 p.i. were double immunolabelled for MBP (green) with Iba1 (red) and PLP (green) with Iba1 (red) and counterstained with DAPI (blue). Insets highlight areas that show engulfment of MBP or PLP by Iba1 + MG/Mφ. Scale bar 100 μm for epifluorescence image and 10 μm for confocal image

To further confirm that the phagocytic phenotype of Iba1 + MG/Mφ was responsible for myelin loss at day 9/10 p.i., confocal imaging of spinal cord sections double immunolabelled for Iba1 and MBP, and Iba1 and PLP, was performed. Amoeboid Iba1 + MG/Mφ were present in regions devoid of MBP and PLP in both WT and NOS2-/- mice (Fig. 4A and B, white arrows) and appeared to move towards the MBP and PLP enriched regions for subsequent engulfment (Fig. 4A and B, insets with yellow arrows indicating engulfment of myelin protein by amoeboid Iba1 + MG/Mφ). Although, it should be noted that the loss of MBP and presence of Iba1 + MG/Mφ was both more abundant in NOS2-/- mice. Thus, it can be concluded that the change in inflammatory milieu of brain due to increased infiltration of MG/Mφ in the absence of NOS2 led to production of phagocytic MG/Mφ phenotypes and early demyelination mediated by these cells.

### NOS2 deficiency leads to severe demyelination at the chronic phase (day 30 p.i.)

**Fig. 5:**
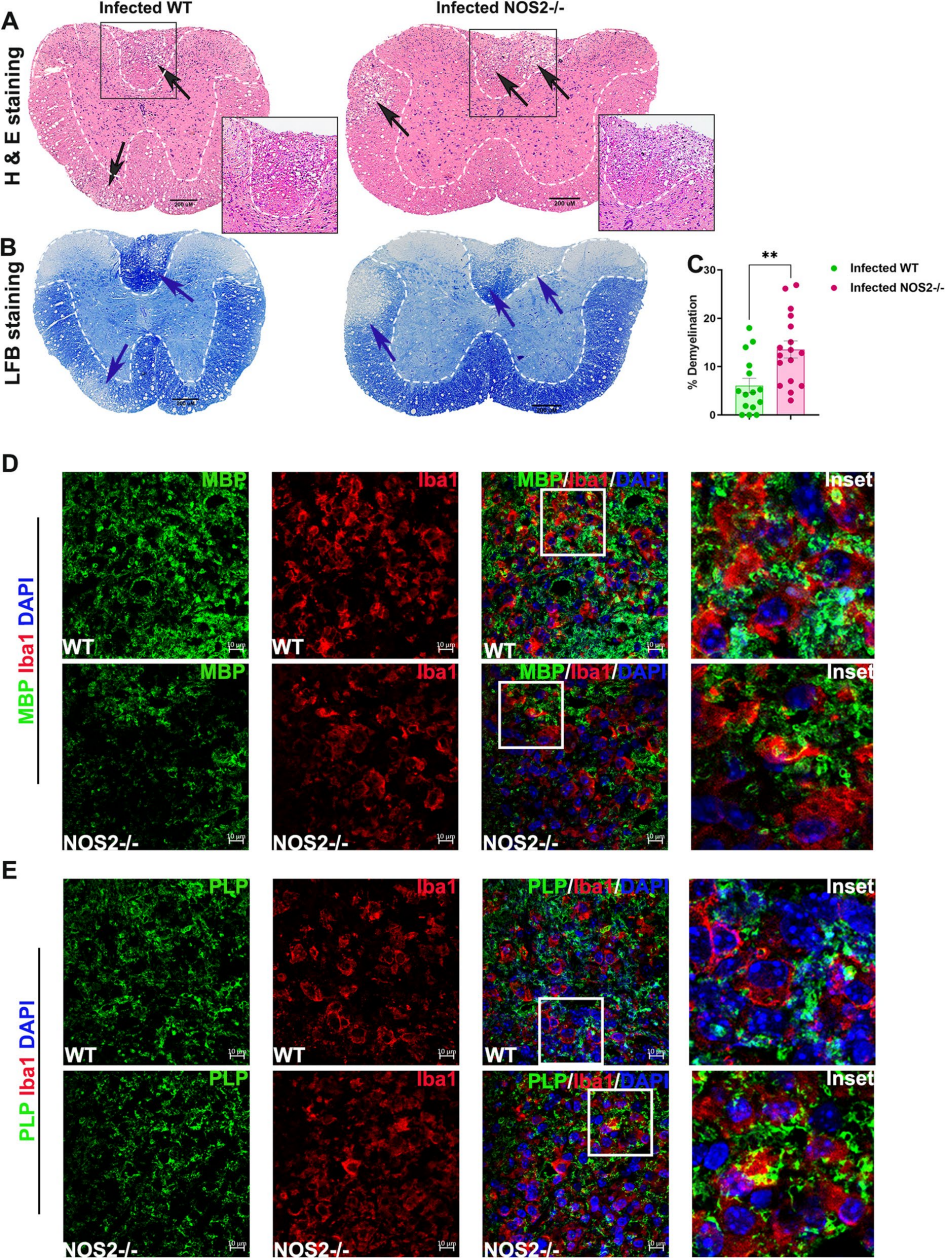
NOS2 deficiency exacerbates the chronic phase (day 30 p.i.) demyelination. 5 µm thick transverse- sections of RSA59 infected (10,000 PFUs) WT and NOS2-/- mice spinal cords were stained for the presence of inflammatory lesions by H & E (**A**), and demyelination by LFB (**B**). Black arrows in (**A**) indicate the regions with myelitis. Regions with black outlined boxes are shown in insets. Blue arrows in (**B**) indicate the demyelinating plaques. Scale bar 200 µm. (**C**) is the quantification of percent demyelination. (n = 5 to 7 and the dots represent all the sections from all the spinal cords. 1 to 6 sections corresponding to different regions of the spinal cord were analysed). *Asterisk represents statistical significance calculated between infected WT and infected NOS2-/- mice using unpaired student’s t test with Welch’s correction. **p value < 0.01. Confocal photomicrographs of demyelinating plaques are represented in (**C**) through (**D**). 5 μm thick transverse spinal cord sections from infected WT and NOS2-/- mice were double immunolabelled for MBP (green) with Iba1 (red) and PLP (green) with Iba1 (red) and counterstained with DAPI (blue). Insets highlight areas that show engulfment of MBP or PLP by Iba1 + MG/Mφ. Scale bar 10 μm

Demyelination in RSA59 infected WT mice is a characteristic of the chronic phase and is a direct consequence of myelin striping by amoeboid microglia [17, 21]. Since NOS2-/- mice showed the presence of prominent demyelinating plaques as early as day 9/10 p.i., the effect of NOS2 on chronic phase demyelination was checked next. H & E, and LFB staining were performed on spinal cord sections from WT and NOS2-/- mice infected at 10,000 PFUs. NOS2-/- mice showed more myelitis (Fig. 5 A black arrows) and demyelination in the spinal cords (Fig. 5 B blue arrows). The demyelination quantified showed a significantly higher percentage in NOS2-/- mice. Confocal staining of areas corresponding to demyelination plaques in parallel sections showing prolific presence of amoeboid Iba1 + MG/Mφ engulfing myelin protein was found in both infected WT and NOS2-/- spinal cord sections as expected at day 30 p.i. (Fig. 5C and D, insets). Although, loss of MBP was comparatively more in infected NOS2-/- (Fig. 5C) spinal cord sections. Thus, it can be concluded that effect of NOS2 was persistent for as long as day 30 p.i. and its absence worsened the demyelination pathology over time.

## Discussion

The role of NOS2 in neurodegeneration has been studied in murine experimental models of MS utilising demyelination-inducing chemicals, peptides, or virus strains, the outcomes of which often depend on differential disease pathology and the role of infiltrating T cells. In the early 2000s Lane and Perlman laboratories showed that absence of NOS2 does not affect demyelination [11, 32]. These studies were carried out incorporating MHV-JHM, a highly neurovirulent strain of MHV as compared to RSA59 [33]. In the neurotoxicant cuprizone-induced demyelination model, on the other hand, absence of NOS2 exacerbated demyelination at early time points [15]. As compared to JHM where mice succumb to death at day 12 p.i. due to severe demyelination, RSA59 infection in WT mice causes prominent demyelination around day 30 p.i. allowing us to dissect the immune responses involved over time. The current study was thus initiated to revisit the role of NOS2 in demyelination using MHV-RSA59 infection as the model.

RSA59 infection in C57BL/6 WT mice led to upregulation of NOS2 mRNA and protein at the acute phase, i.e., day 5/6 p.i. confirming its role in RSA59-induced acute neuroinflammation. Further NOS2 deficiency led to increased mortality and uncommon demyelination pathology at the acute-adaptive transition phase. However, it did not affect viral clearance, which is in line with the above reports. We have previously reported the crucial role of CD4 + T cells in mounting anti-viral immune response against RSA59. In the absence of either CD4 + T cells or their activation marker CD40L which mediates the MG/Mφ-CD4 + T cell crosstalk, the virus persisted in the CNS. This in turn lead to continual activation of MG/Mφ concurrent with high expression of phagocytic markers to eliminate both the debris and virus-infected cells resulting in severe chronic demyelination as a bystander effect [23, 24]. In addition, ligation of CD40 on microglial cells has been reported to induce NOS2 expression [34]. In the absence of NOS2 the crosstalk between MG/Mφ and CD4 + T cell was not affected as evident from successful viral clearance and comparable CD4 + T cell numbers in infected NOS2-/- mice, however there was an increased infiltration of macrophages and neutrophils in infected NO2-/- mice brains at the acute-adaptive transition phase. The infected NOS2-/- brains also showed higher levels of inflammatory markers TNFα, IFNγ and Ifit2 which could be attributed to the increased infiltration of macrophages and neutrophils though the inflammatory status needs to be reconfirmed at protein expression level as well. Many studies have reported direct role of NO in inhibiting leukocyte recruitment by reducing the leukocyte-endothelial interactions [35]. Thus, lower levels of NO in NOS2-/- mice could be one of the reasons for high macrophage and neutrophil infiltration. A slightly similar alteration in myeloid cell infiltration was also reported in a relatively recent study in the experimental autoimmune encephalomyelitis (EAE) model of MS where it was showed that in the absence of NOS2 there was high recruitment of pathogenic CD11b + F4/80 − Gr-1 + cells and low infiltration of regulatory CD11b + F4/80 − cells in the brain during the priming phase and in the spinal cord during the effector phase [36]. This indicated that effect of NOS2 was phase-dependent which is also apparent in the current study. NOS2 production is highest at the acute phase (day 5/6 p.i.), its deficiency however did not affect CNS pathology during acute neuroinflammation (day 5/6 p.i.) but resulted in early demyelination at the acute-adaptive transition phase (day 9/10 p.i.) implying that the system adopted auxiliary mechanisms which could not be sustained over time. Demyelination pathology at the acute-adaptive transition phase in the spinal cords of NOS2-/- mice was accompanied by amoeboid Iba1 + MG/Mφ. Early studies by Wu and Perlman in MHV-JHM model have shown a correlation of increase in the number of F4/80 + macrophages with demyelination and showed the presence of distinct activated ameboid F4/80 + macrophages in the demyelinating plaques [33]. The early demyelination observed in the RSA59 infected NOS2-/- mice was accompanied by significant upregulation of MG/Mφ phagocytic markers CD206, TREM2 and Arg1 in the CNS confirming the anti- inflammatory phagocytic phenotype of MG/Mφ [37–41]. The phagocytic markers were not further verified at protein expression level however, confocal imaging of the demyelinating plaques confirmed considerably more loss of MBP due to engulfment by Iba1 + MG/Mφ in infected NOS2-/- spinal cord sections. A lucid study in the EAE induced model of MS targeted the expression of NOS2 and Arg1 by mononuclear phagocytes showing that the cells specify and adapt to their phenotype locally guided by the CNS-derived signals and can change their individual phenotypes over time accordingly [42]. Another aspect of regulation of polarization in macrophages was shown by Van den Bossche et al. [43] where inhibition of NOS2 led to the rescue of NO suppressed mitochondrial respiration and enhanced inflammatory to anti-inflammatory phenotypic repolarization of macrophages. This proved that the local redox and immune milieu is important in deciding the fate of the cells and thereby the pathological outcome. It can thus be concluded that changes in brain inflammatory milieu due to more influx of macrophages and neutrophils led to early progression of MG/Mφ into an amoeboid phagocytic phenotype responsible for early demyelination in the absence of NOS2. Though anti-inflammatory phagocytic phenotype has been shown to be beneficial in various inflammatory diseases [44], persistent activation could damage the healthy tissue as observed by RSA59-induced early demyelination in this study which worsened by day 30 p.i. at which point majority of the myelin MBP was stripped off from the white matter axons. Current study thus suggests that the absence of NOS2 led to the loss of regulation in the recruitment of macrophages and neutrophils leading to enhanced inflammation which potentially caused the increased disease severity and mortality in the mice.

## Conclusion

Contrary to the previously reported pathogenic role of NOS2 in MS and related autoimmune animal models, we show that β-coronavirus-RSA59 infected NOS2 deficient mice exhibit exacerbated disease with accelerated demyelination. This was accompanied by increased accumulation of neutrophils and macrophages and a high prevalence of phagocytic MG/Mφ. We thus show a protective role of NOS2 in RSA59-induced demyelination and the study adds to the limited knowledge of the role of NOS2 in modulating the demyelination process in experimental animal models of MS.

## Supplementary Information

**Additional file 1**. Supplementary images for the main text.

## Data Availability

The datasets supporting the conclusions of this article are included within the article and its supporting information.

## Abbreviations

MS: Multiple sclerosis
CNS: Central nervous system
NOS2: Inducible nitric oxide synthase
MHV: Mouse hepatitis virus
WT: Wildtype
p.i.: Post infection
MG/Mφ: Microglia/macrophage
TNFα: Tumour necrosis factor α
IFNγ: Interferon γ
Ifit2: Interferon induced protein with tetratricopeptides 2
CD206 or MRC1: Mannose receptor C type 1 protein
TREM2: Triggering receptor expressed on myeloid cells 2
Arg1: Arginase 1
NO: Nitric oxide
NOS: Nitric oxide synthase
MFI: Median fluorescent intensity
GAPDH: Glyceraldehyde-3-phosphate dehydrogenase
N gene: Nucleocapsid gene
LFB: Luxol fast blue staining
MBP: Myelin basic protein
PLP: Proteolipid protein
EAE: Experimental autoimmune encephalomyelitis
