# Supplementary material for: Inducible nitric oxide synthase deficiency promotes murine-β-coronavirus induced demyelination
Source: Virol J. 2023 Mar 25;20:51. doi: 10.1186/s12985-023-02006-1 (PMC10039690; doi:10.1186/s12985-023-02006-1)
Supplement: Supplementary file 1 — Additional file 1. Supplementary images for the main text. [file 12985_2023_2006_MOESM1_ESM.docx]

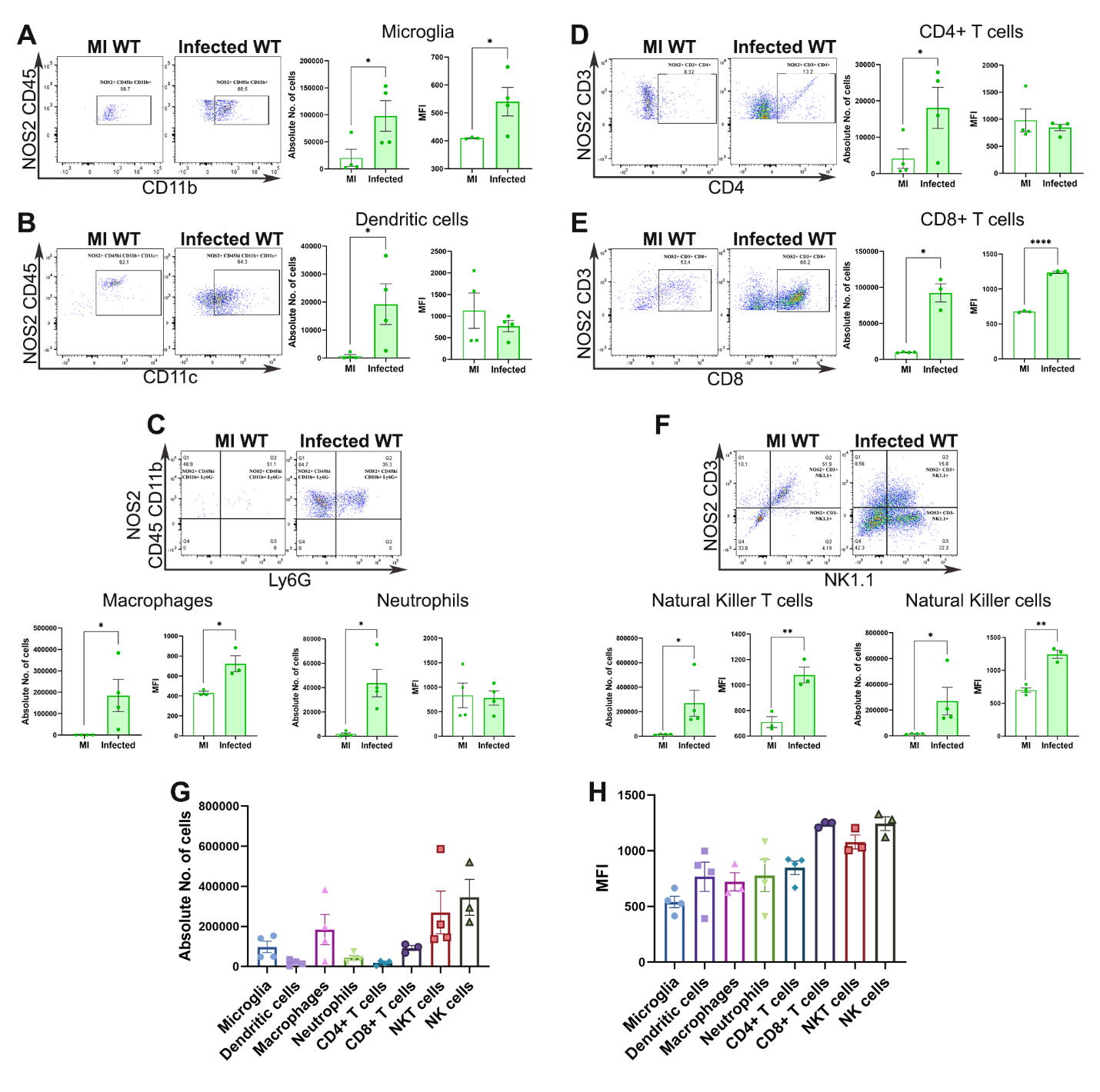
**Fig S1. NOS2 expression is significantly upregulated in myeloid and lymphoid immune cell subsets.**

Brains were harvested from mock and infected WT mice (10000 PFUs) on day 5 p.i. for flow cytometry analysis and stained for NOS2, CD45, CD11b, CD11c, Ly6G, CD3, CD4, CD8 and NK1.1. NOS2+ cells were gated from live cell populations gated from singlets. Subsequent immune cell subsets were gated from NOS2+ cells and their absolute numbers and NOS2 MFI were compared between mock and infected WT groups. NOS2+ infiltrating myeloid cells were gated from NOS2+ CD45hi while NOS2+ resident microglia were gated from NOS2+ CD45lo. Lymphoid cell subsets expressing NOS2 were gated from NOS2+ CD3+ for peripheral T cells and NOS2+ CD3- for NK cells. Representative dot plots and graphical representation of number of cells and NOS2 MFI are given for NOS2+ CD45lo CD11b+ microglia (A), NOS2+ CD45hi CD11C+ dendritic cells (B), NOS2+ CD11b+ Ly6G- macrophages and NOS2+ CD11b+ Ly6G+ neutrophils (C), NOS2+ CD3+ CD4+ T cells (D), NOS2+ CD3+ CD8+ T cells (E), NOS2+ CD3+ NK1.1 (NKT) cells and NOS2+ CD3- NK1.1 (NK) cells (F). Dot plots comparing NOS2+ cells in different cell subsets is represented in (G) and dot plot comparing NOS2 MFI in different cell subsets is represented in (H). Results were expressed as Mean ± SEM. *Asterisk represents statistical significance calculated using unpaired student’s t test with Welch’s correction, p< 0.05 was considered as significant. *p< 0.05, **p< 0.01, and ****p< 0.0001. n= 3 to 4 mice per group


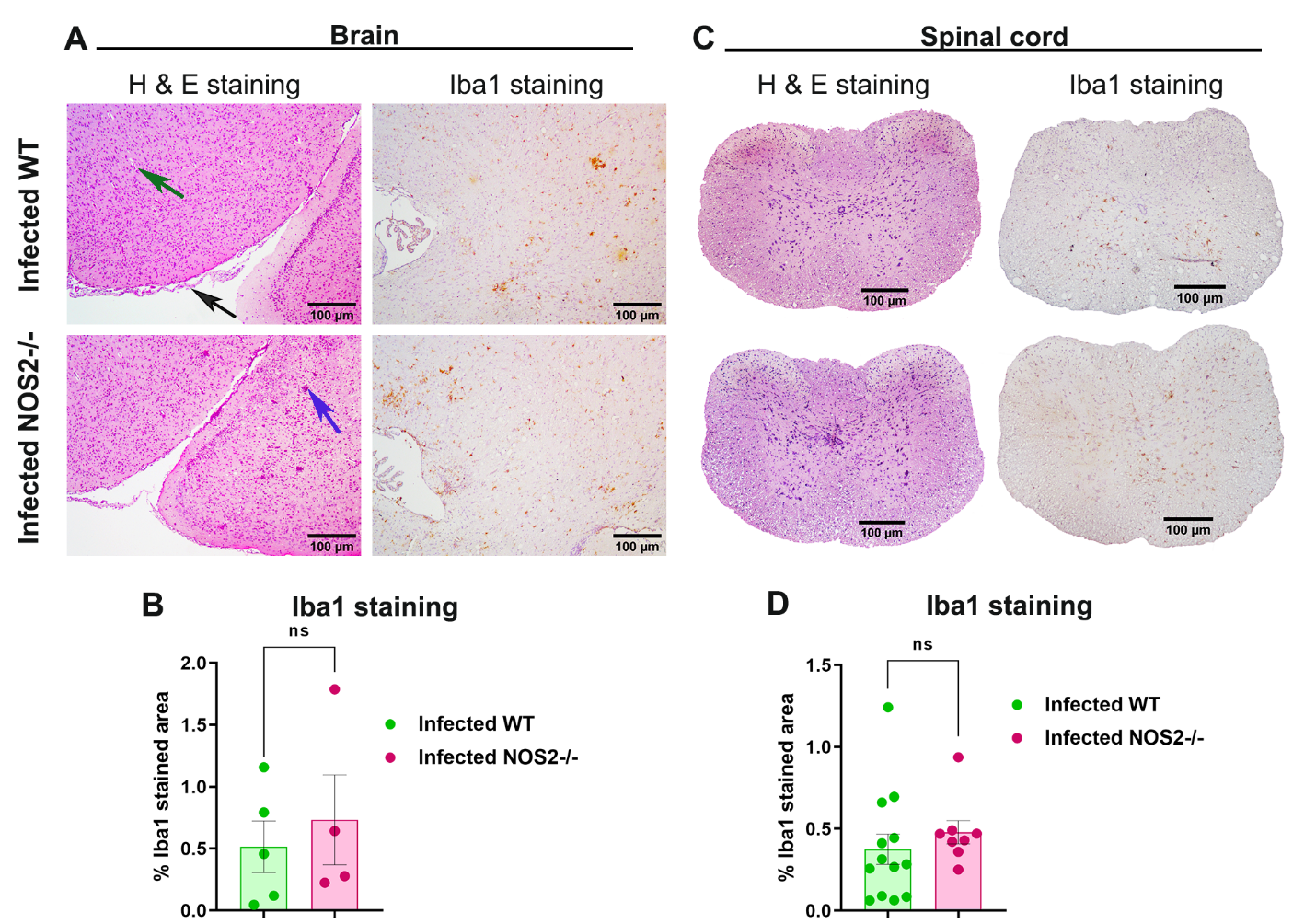


**Fig S2. NOS2 deficiency did not affect the acute phase CNS histology.**

Brain and spinal cords were harvested from RSA59 infected (20000 PFUs) WT and NOS2-/- mice at day 5/6 p.i. 5µm thick sagittal sections of brain (E) and 5µm thick transverse sections of spinal cord (F) were stained for the presence of inflammatory lesions by H & E and activated MG/Mφ by Iba1. Scale bar 100µm. (G) and (H) is the quantification of percent Iba1 stained area of brain and spinal cord respectively. Results were expressed as Mean ± SEM. Statistical significance was calculated between infected WT and infected NOS2-/- mice using unpaired student’s t test with Welch’s correction. p<0.05 was considered significant. n= 4 to 6 mice per group. *


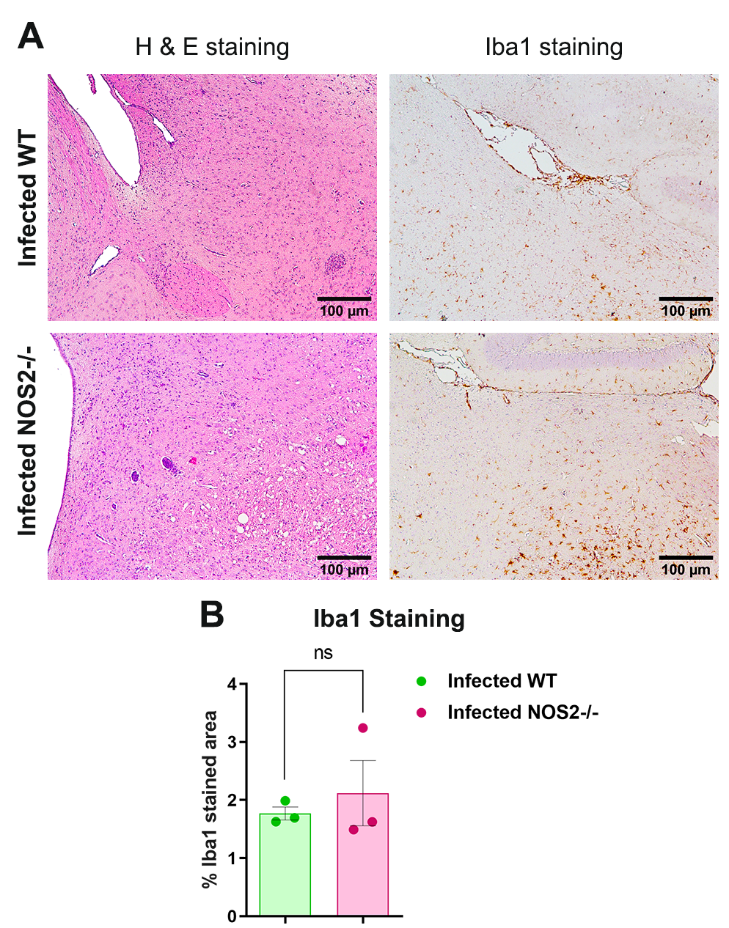


**Fig S3. NOS2 deficiency did not affect brain pathology at the acute-adaptive transition phase (day 9/10 p.i.).**

WT and NOS2-/- mice were infected with 20000 PFUs of RSA59. The brain tissues from mice sacrificed at day 9/10 p.i. were processed and sagittal sections of 5µm thickness were used to perform routine histopathology. (A) shows H&E staining and Iba1 staining of infected brains at day 9/10 p.i. Characteristic pathology of acute phase inflammation, i.e., meningitis (black arrow), vascular cuffing (green arrow), and microglial nodules (blue arrow) are indicated. Quantification of Iba1 staining at day 9/10 p.i. is shown graphically in B. Statistical significance was calculated using unpaired student’s t – test with Welch’s correction, p<0.05 was considered significant. n= 3 to 5.

**
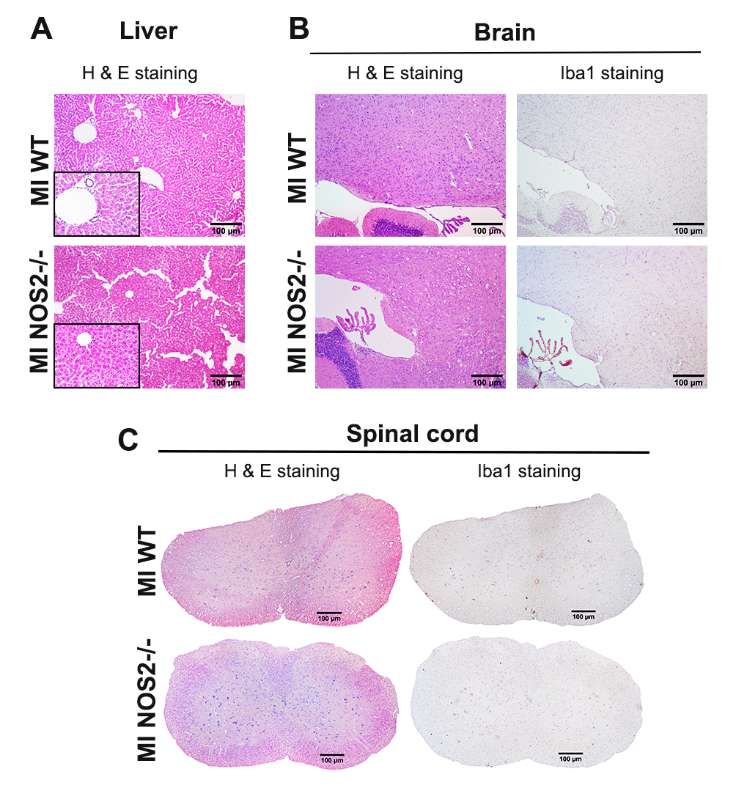
**

**Fig S4. NOS2 deficiency did not cause any pathology in mock infected mice at the acute phase (day 5 p.i.).**

WT and NOS2-/- mice injected with PBS-BSA intracranially were used as mock controls. 5µm thin sections of liver (A), 5µm thick sagittal sections of brain (B), and 5µm thick transverse sections of spinal cords (C) from mock infected WT and NOS2-/- mice were stained with H&E (liver, brain, and spinal cord) and Iba1 antibody (brain and spinal cord), at day 5 p.i. The images shown are representative. The tissues exhibited no significant inflammation. n= 3.


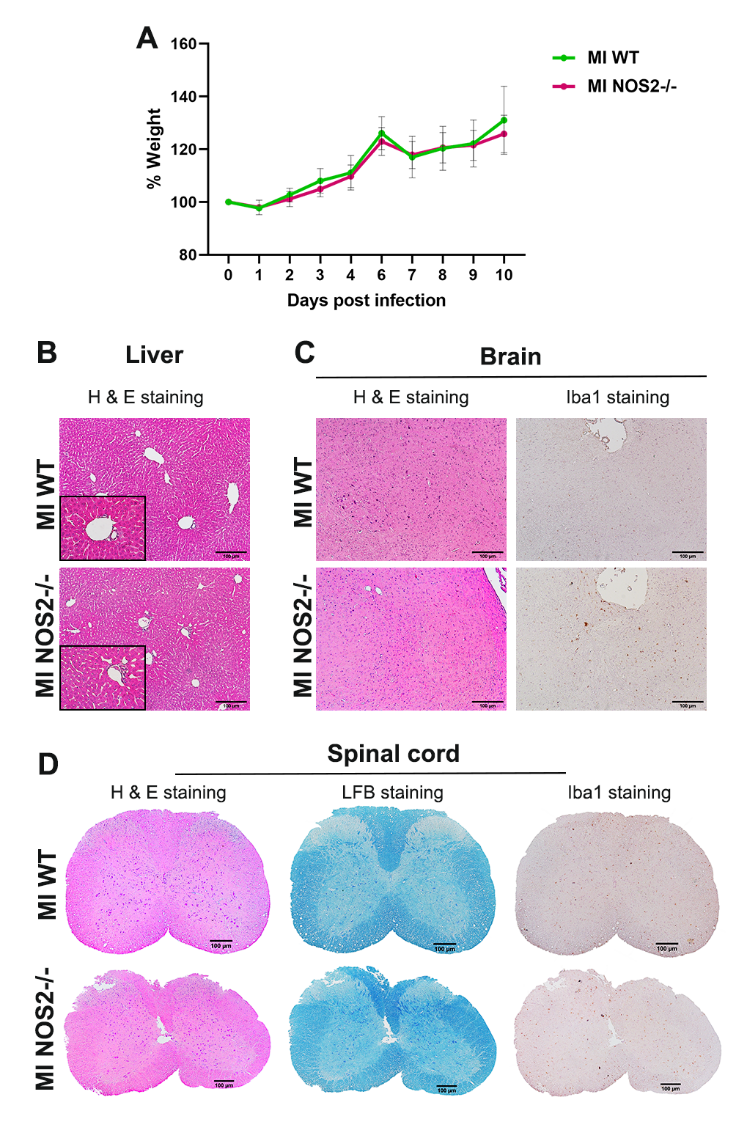


**Fig S5. NOS2 deficiency does not cause any physiological or pathological alterations in mock infected mice at the acute-adaptive transition phase (day 9/10 p.i.).**

WT and NOS2-/- mice were infected with PBS-BSA, monitored for weight change (A) daily and sacrificed at day 10 p.i. 5µm thin sections of liver (A), 5µm thick sagittal sections of brain (B) and 5µm thick transverse sections of spinal cord (C) were subjected to H&E staining (liver, brain, spinal cord), LFB staining (spinal cord) and Iba1 staining (brain and spinal cord). No differences were observed in the weight change and histopathology between WT and NOS2-/- mice. n= 3 to 5.
